# Acute stress symptoms in general population during the first wave of COVID lockdown in Italy: Results from the COMET trial

**DOI:** 10.1002/brb3.3314

**Published:** 2023-11-21

**Authors:** Claudia Carmassi, Gaia Sampogna, Matteo Di Vincenzo, Salvatore Cipolla, Claudia Toni, Umberto Albert, Giuseppe Carrà, Francesca Cirulli, Bernardo Dell'Osso, Sara Fantasia, Maria Giulia Nanni, Virginia Pedrinelli, Maurizio Pompili, Gabriele Sani, Alfonso Tortorella, Umberto Volpe, Andrea Fiorillo

**Affiliations:** ^1^ Department of Clinical and Experimental Medicine University of Pisa Pisa Italy; ^2^ Department of Psychiatry University of Campania “L. Vanvitelli” Naples Italy; ^3^ Department of Medicine, Surgery and Health Sciences University of Trieste and Department of Mental Health, Azienda Sanitaria Universitaria Giuliano Isontina–ASUGI Trieste Italy; ^4^ Department of Medicine and Surgery University of Milan Bicocca Milano Italy; ^5^ Center for Behavioral Sciences and Mental Health National Institute of Health Rome Italy; ^6^ Neuroscience Research Center, Department of Biomedical and Clinical Sciences and Aldo Ravelli Center for Neurotechnology and Brain Therapeutic University of Milan Milano Italy; ^7^ Department of Psychiatry and Behavioral Sciences Stanford University Stanford California USA; ^8^ Institute of Psychiatry, Department of Neurosciences and Rehabilitation University of Ferrara Ferrara Italy; ^9^ Department of Neurosciences, Mental Health and Sensory Organs, Faculty of Medicine and Psychology Sapienza University of Rome Rome Italy; ^10^ Department of Neuroscience, Section of Psychiatry University Cattolica del Sacro Cuore Rome Italy; ^11^ Department of Neuroscience, Sensory organs and Thorax, Department of Psychiatry Fondazione Policlinico A. Gemelli IRCCS Rome Italy; ^12^ Department of Psychiatry University of Perugia Perugia Italy; ^13^ Clinical Psychiatry Unit Department of Clinical Neurosciences Università Politecnica delle Marche Ancona Italy

**Keywords:** acute stress, acute stress symptoms, ASD, COVID‐19, lockdown, post‐traumatic stress symptoms, PTSD

## Abstract

**Background:**

The coronavirus disease of 2019 (COVID‐19) pandemic is an unprecedented traumatic event that has severely impacted social, economic, and health well‐being worldwide. The COvid Mental hEalth Trial was specifically designed to evaluate the impact of the COVID‐19 pandemic and its containment measures on the mental health of the Italian general population in terms of COVID‐19‐related acute stress disorder (ASD) symptoms.

**Methods:**

The present cross‐sectional study is based on an online survey carried out in the period March–May 2020. Italian general adult population was invited to compile an anonymous survey, which included the severity of acute stress symptoms scale/National Stressful Events Survey Short Scale to investigate the occurrence and severity of ASD symptoms.

**Results:**

The final sample consisted of 20,720 participants. During the lockdown, subjects with pre‐existing mental health problems reported a statistically significant higher risk of acute post‐traumatic symptoms compared to the general population (*B*: 2.57; 95% CI:2.04–3.09; *p* < .0001) and health care professionals (*B*: .37; 95% CI: .02–0.72; *p* < .05). According to multivariate regression models, the levels of acute post‐traumatic symptoms (*p* < .0001) were higher in younger and female respondents. Social isolation and sleep disorder/insomnia represented positive predictors of acute stress (*B* = 3.32, 95% CI = 3.08–3.57).

**Conclusions:**

Concerns about the risk of infection as well as social isolation caused a higher incidence of acute post‐traumatic stress symptoms that may predict the subsequent development of post‐traumatic stress disorder symptoms in the long term.

## Graphical abstract

In our paper, we analyzed the impact of COVID‐19 pandemic on the general population in Italy, during the first phase of the lockdown. In particular, data were collected from more than 20,000 persons from the general population using the severity of acute stress symptoms scale, together with several other validated assessment tools.

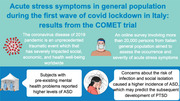

## 1 INTRODUCTION

The coronavirus disease of 2019 (COVID‐19) is a highly contagious infectious disease caused by a novel coronavirus, named severe acute respiratory syndrome coronavirus 2 (SARS‐CoV‐2). It was first identified in Wuhan (China) in December 2019. On January 30th, 2020, the World Health Organization declared the new coronavirus a public health emergency of international concern and the outbreak of SARS‐CoV‐2 was declared a pandemic in March 2020 (Eurosurveillance Editorial Team, 2020; Mei et al., 2021). On November 2, 2023, the virus had infected more than 770 million people and killed almost 7 million people worldwide (Health Emergency Dashboard, 2023).

In Europe, Italy was the first country to be heavily affected by the pandemic, which led to the imposition of a nationwide lockdown, allowing only essential activities. These containment measures applied from March 8th, 2020 to May 3rd, 2020 (known as “Phase 1” of the pandemic emergency), followed by a gradual reopening of services (“Phase 2”), affecting approximately 60 million people. During the first wave of the pandemic (March–April 2020), although strict containment measures were issued by the Italian Prime Minister, the spread of SARS‐CoV‐2 in Italy was extremely uneven geographically, with Northern regions—where the first cases had been detected—being the most affected in terms of number of severe cases and deaths, followed by Central and Southern regions (De Natale et al., 2020; Pluchino et al., 2021).

The link between viral epidemics and mental health problems is known since 1918, with the Spanish flu pandemic (Menninger, 1920), due to some direct actions (i.e., neuroinvasive capabilities of viruses, Desforges et al., 2019; Mazza et al., 2020) or to indirect factors (i.e., living in a pandemic or surviving a severe disease, Deng et al., 2021; Fiorillo & Gorwood, 2020; Giannopoulou et al., 2021).

Numerous studies have documented an unprecedented increase in mental health problems, particularly anxiety and depression, correlated to the pandemic and its associated containment efforts. Moreover, stressful experiences, from the outbreak itself or the subsequent social containment measures adopted to face the outbreak (e.g., lockdown and travel restrictions) occurred in a very short period, would have induced acute stress disorder (ASD) symptoms (Ye et al., 2020). ASD refers to a specific mental disorder occurring within 2–28 days following a major traumatic experience, such as actual or threatened death or serious injury (Bryant et al., 1998), which is characterized by a constellation of acute distress symptoms such as intrusive distress memories and hypervigilance until dissociative reactions. In addition, ASD was found to be one of the key precursors for post‐traumatic stress disorder (PTSD) (Bryant, 2022; Zhou et al., 2015). Tsur et al. (2021) in a study carried out in China, Israel, and Switzerland found that the overall clinical picture of acute stress symptoms is universal, highlighting the central role of hyperarousal symptoms.

Previous outbreaks of infectious disease (e.g., SARS, Ebola, and MERS) have shown the detrimental influences of disease‐related stress on emerging acute distress (Greenberg & Rafferty, 2021; Lau et al., 2016; Lee et al., 2018; Zheng et al., 2005). Similarly, the COVID‐19 pandemic itself and lockdown measures can also induce similar problems in the population involved. However, recent studies showed heterogeneity in the pandemic response depending on individual characteristics and area‐specific factors (Ahrens et al., 2021; Coleman et al., 2022; Fiorillo & Gorwood, 2020; Holt‐Lunstad, 2021; Kestel, 2022; Pierce et al., 2020; Pompili et al., 2022; Sampogna, Del Vecchio et al., 2021; Howard et al., 2022; Shevlin et al., 2023; Steger, 2022; Wang et al., 2021; Tzur Bitan et al., 2022). These data are consistent with those found in other emergency situations; for example, in earthquakes, a correlation has been found between the degree of psychopathology and the distance from the epicenter and, therefore, the degree of exposure to the event (Dell'Osso et al., 2013; Turner, 2022). This aspect is particularly important for the Italian population because, as mentioned above, the pandemic in Italy had different epidemiological characteristics depending on the geographic area.

However, some authors have pointed out that the pandemic per se should not be considered a “trauma” for most people, as the term “trauma” usually defines actual or threatened death or serious injury (Norrholm et al., 2021). It should be that the pandemic experience varied greatly among individuals, with someone facing life‐threatening illness or loss of close ones, whereas others were less impacted from the consequences of the pandemic and its containment measures.

Investigating the factors associated with ASD would be beneficial for the prevention of subsequent occurrence of PTSD. Moreover, several studies carried out during the different phases of the pandemic worldwide showed worsening of psychiatric symptoms of those already affected by severe mental health problems.

Therefore, the COvid Mental hEalth Trial (COMET) was specifically set up to evaluate the impact of the COVID‐19 pandemic and its containment measures on the mental health of the Italian general population. However, little is known about the COVID‐19‐related ASD. In this regard, this study aims to report on the expression of acute stress symptoms in the Italian population and to identify individual and area‐specific factors that influenced its degree.

## 2 METHODS

The COMET network includes nine Italian University centers which promoted a study targeting the Italian adult general population during the first wave of the COVID pandemic in Italy. Given the restriction measures, the study was conducted online between March 30 and May 2, 2020. The online survey was developed on the EUSurvey web platform, which is hosted by the European Commission (2013). The survey took approximately 30 min (range 15–45 min) to be completed.

The promotion and dissemination of the survey included a multistep procedure: (1) email invitation sent to health professionals and their patients; (2) publication on social media channels (Facebook, Twitter, and Instagram); (3) dissemination through mailing lists of national psychiatric associations; (4) involvement of national associations of stakeholders (e.g., associations of users/careers); (5) official communication channels (e.g., university and hospital websites).

Being an online survey, the snowball sampling procedure was adopted; therefore, no strict inclusion/exclusion criteria (except age) were defined. This methodological choice allowed to obtain a large sample of the Italian population and to evaluate the effect of the studied variables on the outcome measures. Further details about the study protocol and the results about primary outcome have been published elsewhere (Fiorillo et al., 2020; Giallonardo et al., 2020; Menculini et al., 2021; Sampogna, Giallonardo et al., 2021). The study has been approved by the Ethical Review Board of the University of Campania “L. Vanvitelli” (protocol number: 0007593/i).

Respondents’ sociodemographic (e.g., gender, age, and working and housing condition) and clinical variables (e.g., having a previous physical or mental disorder and use of illicit drugs or medications) were collected through a dedicated questionnaire.

For the purposes of the present paper, specifically focused on the traumatic impact of the exposure to the strict lockdown measure, the main outcome measure is the global score at the Severity‐of‐Acute‐Stress‐Symptoms‐Adult scale/National Stressful Events Survey Short Scale (NSESSS).

The primary outcome of the present paper was the Severity‐of‐Acute‐Stress‐Symptoms‐Adult scale/NSESSS, which has been used to assess the presence of traumatic stress symptoms. The NSESSS consists of nine items rated on a five‐point scale (from 0 = Not at all to 4 = Extremely). The total score ranges from 0 to 28, with higher scores indicating a greater severity of ASDs (Kilpatrick et al., 2013). Respondents are asked to fill in the questionnaire considering the past seven (7) days as time‐frame for each of the none items included in the scale. The Cronbach alpha is of good level, with a value of .901 (LeBeau et al., 2014).

Other validated and reliable assessment tools have been included in the online survey and all details are available in the study protocol (Giallonardo et al., 2020). In particular, in the present study, the results on the Impact of Event Scale (IES)—short version, which measures the traumatic reactions in people who have experienced traumatic events, have been considered. Each item is rated on a 5‐point scale ranging from 0 (not at all) to 5 (often). The IES evaluates the dimensions of intrusion, avoidance, and alteration in arousal (Thoresen et al., 2010). The IES has a Cronbach alpha rating of .79–.91 (intrusion) and .82–.90 (avoidance).

The Depression Anxiety Stress Scales (DASS‐21), for assessing the general distress on a tripartite model of psychopathology, was used (Lovibond, 1995). It consists of 21 items grouped in three subscales: depression, anxiety, and stress. The depression scale assesses dysphoria, hopelessness, devaluation of life, self‐deprecation, lack of interest/involvement, anhedonia, and inertia. The anxiety scale assesses autonomic arousal, skeletal muscle effects, situational anxiety, and subjective experience of anxious affect. The stress scale evaluates the levels of chronic nonspecific arousal. It assesses difficulty relaxing, nervous arousal, and being easily upset/agitated, irritable/over‐reactive, and impatient. Each item is rated on a 4‐level Likert scale, from 0 (never) to 3 (almost always). The total score is calculated by adding together the response value of each item, with higher scores indicating more severe levels of depressive, anxiety, and stress symptoms. Cronbach's alphas of the three subscales of DASS are .81 (depressive symptoms), .89 (anxiety symptoms), and .78 (stress symptoms), respectively.

The General Health Questionnaire (GHQ)—12 items version explores participants’ mental health status through six positively worded items (e.g., “Have you been able to concentrate?”) and six negatively worded items (e.g., “Have you lost much sleep over worry?”). The standard scoring method recommended by Goldberg for the need of case identification is called “GHQ method.” Scores for the first two types of answers are “0” (positive) and for the other two are “1” (negative). Threshold ≥4 at GHQ identifies people with a probability >80% of having a mental health problem (Goldberg et al., 1997). The Cronbach's alpha of the GHQ‐12 is .9.

The Insomnia Severity Index (ISI) was used for evaluating the presence of sleep‐related disorder. It includes seven items rated on a 5‐level Likert scale (from 0 to 4), with a total score ranging from 0 to 28 (Morin et al., 2011). The Cronbach's alpha is .92.

The UCLA loneliness scale—short version is an eight‐item scale designed to measure subjective feelings of loneliness, as well as feelings of social isolation. Each item is scored on a 4‐level Likert scale from 0 = never to 3 = often (Hays & Di Matteo, 1987). The Cronbach's alpha of the scale is .84.

The Suicidal Ideation Attributes Scale (SIDAS) consists of five items assessing frequency, controllability, closeness to attempt, level of distress associated with suicidal thoughts, and impact on daily functioning. Each item is assessed on a 10‐level Likert scale, with a total score ranging from 0 to 50. In case of scoring “0—Never” to the first item, all other items are skipped, and the total score is zero. The presence of any suicidal ideation is considered indicative of risk for suicidal behavior, whereas a cut‐off of 21 is used to indicate high risk of suicidal behavior (Van Spijker et al., 2014). The Cronbach alpha is .91.

The Connor–Davidson resilience scale is used for evaluating the levels of resilience. It includes 10 items rated on a 6‐level Likert scale and is subdivided into the following five factors: (1) personal competence, high standards, and tenacity; (2) trust in one's instincts, tolerance of negative affect, and strengthening effects of stress; (3) positive acceptance of change and secure relationships; (4) control; and (5) spiritual influences. Higher values indicate higher levels of resilience (Connor & Davidson, 2003). The Cronbach alpha is .84.

Coping strategies have been evaluated using the Brief‐COPE, consisting of 28 items grouped in 14 subscales (Carver, 1997). Each item is rated on a 4‐level Likert scale from 0 = “I have not been doing this at all” to 3 = “I have been doing this a lot.” Coping strategies are grouped into maladaptive strategies, including denial, venting, behavioral disengagement, self‐blame, self‐distraction, and substance abuse, and adaptive coping strategies, which include emotional support, use of information, positive reframing, planning, and acceptance. Two other subscales include religion and humor. The Cronbach alpha is .70.

## 3 DATA ANALYSIS

Descriptive statistics were performed in order to describe the sociodemographic and clinical characteristics of the global sample and as well as any difference between patients with pre‐existing mental disorders and the remaining sample.

In order to evaluate factors associated with the severity of traumatic symptoms in the global sample, a multivariate linear regression model has been implemented, using the total score at the SASS scale as main outcome measure.

Several confounding variables have been included in the model, such as being infected by COVID‐19, having a pre‐existing mental disorder, being a health care professional, living in different geographic areas, coping strategies, perceived loneliness, general health, insomnia symptoms as well as number of COVID cases, and mortality COVID‐related rate. Moreover, analyses have been controlled for individual factors, such as age and gender, but effect estimates for those factors have not been interpreted as suggested by Westreich and Greenland (2013).

We used a propensity score in order to adjust for the likelihood of participants of being exposed to COVID‐19 infection in each week. The propensity score represents a valid approach in observational studies in order to obtain an unbiased estimate of the effect of the exposure to a variable. The propensity score is the probability of having the considered outcome conditional on observed baseline covariates (Austin et al., 2018). This methodological choice has been adopted considering that the propensity score produces a better adjustment for differences at baseline rather than simply including potential confounders in the multivariable models. The propensity score was calculated using age, gender, socioeconomic status, and living in a severely impacted area as independent variables.

Furthermore, in order to evaluate the impact of the lockdown duration and other related containment measures on the primary outcomes, the categorical variable “week” was also included in the regression models. The models were adjusted for the rate of new COVID‐19 cases and COVID‐19‐related mortality during the study period, as well as for several sociodemographic characteristics, such as gender, age (managed as categorical variable), occupational status, having a physical comorbid condition, levels of perceived loneliness, health status, taking any pharmacological agent for comorbid mental health conditions, type of adopted coping strategies, levels of perceived loneliness, levels of general health, and presence of insomnia symptoms. This statistical approach has been used also in previous papers based on COMET dataset (Fiorillo et al., 2020; Giallonardo et al., 2020; Menculini et al., 2021; Sampogna, Giallonardo et al., 2021). Missing data were handled using the multiple imputation approach, although the rate of missing data was < 1%. For the outcome variable considered in the paper the rate was < .1%. All other variables were managed as previously reported.

Statistical analyses were performed using the Statistical Package for Social Sciences, version 26.0 and STATA, version 15. For all analyses, the level of statistical significance was set at *p* < .05.

## 4 RESULTS

The global sample consisted of 20,720 people; 5.5% (*N* = 1133) of them had pre‐existing mental disorders (Table 1).

**TABLE 1:** Sociodemographic and clinical characteristics of the sample (*N* = 20,720).

Age, years, mean ± SD	40.4 ± 14.3
Age groups, % (*N*)	
18–24‐year old	15.2 (3151)
25–55‐year old	65.2 (13,514)
55–64‐year old	14.0 (2904)
Over 65‐year old	5.6 (1151)
Gender, F, % (*N*)	71 (14,720)
Living with partner, yes, % (*N*)	52.2 (10,808)
University degree, yes, % (*N*)	62 (12,844)
Employed, yes, % (*N*)	70 (14,518)
Lost job due to the pandemic, yes, % (*N*)	6.3 (1302)
Are you practicing smart working, yes, % (*N*)	34.2 (7089)
Spending more time on Internet, yes, % (*N*)	80.1 (16,598)
Any comorbid physical condition(s), yes, % (*N*)	14.5 (3012)
Any mental health problem(s), yes, % (*N*)	5.5 (1133)
Have you been infected by COVID‐19, yes, % (*N*)	1.4 (296)
Have you been isolated due to COVID‐19 infection, yes, % (*N*)	1.5 (316)
Have you been in contact with someone affected by COVID‐19, % (*N*)	4.2 (866)
Clinical characteristics	5.6 ± 1.6
General Health Questionnaire—global score, mean ± SD (range: 0–12)	10.7 ± 8.2
Obsessive Compulsive Inventory—global score, mean ± SD (range: 0–72)	9.8 ± 5.2
Insomnia Severity Index, mean ± SD (range: 0–28)	4.9 ± 6.6
Suicidal Ideation Attributes Scale (SIDAS), mean ± SD (range: 0–50)	6.0 ± 4.9
Severity of Acute Stress Symptoms‐Adult, mean ± SD (range: 0–28)	
Impact of Event Scale, mean ± SD (range: 0–5)	
Intrusion	1.1 ± 1.9
Avoidance	2.3 ± 2.0
Hyperarousal	2.5 ± 1.9
Loneliness, mean ± SD (range: 0–24)	19.1 ± 3.6
Coping strategies, mean ± SD (range: 1–4)	
*Maladaptive strategies*	
Self‐distraction	2.7 ± 0.8
Denial	1.5 ± 0.7
Venting	2.7 ± 0.8
Behavioral disengagement	1.6 ± 0.6
Self‐blame	2.4 ± 0.8
Substance use	1.2 ± 0.5
*Adaptive strategies*	
Acceptance	3.1 ± 0.7
Active	2.9 ± 0.8
Emotional support	2.4 ± 0.8
Use of information	2.4 ± 0.8
Positive reframing	2.3 ± 0.7
Planning	3.0 ± 0.8
*Other*	
Religion	1.9 ± 0.9
Humor	2.1 ± 0.8
Post‐Traumatic Growth Inventory, mean ± SD (range: 0–10)	
Personal strength	2.1 ± 3.4
Spiritual change	3.7 ± 2.9
Appreciation for life	6.4 ± 3.2
Relating to others	5.3 ± 1.6
New possibilities	5.8 ± 1.6
Connor—resilience Scale, mean ± SD (range: 0–40)	31.3 ± 10.4
Multidimensional scale of perceived social support, mean ± SD (range: 4–28)	
Family support	21.1 ± 6.7
Friends support	20.3 ± 6.5
Support from other relevant ones	22.3 ± 6.7

In the global sample, the most frequent dimensional symptoms of trauma were feeling emotionally upset (in 42.40% of cases) and avoidant behaviors (in 35.11% of cases). Almost 20% of participants reported to have flashbacks or feelings of detachment from the reality (Table 2).

**TABLE 2:** National Stressful Events Survey Acute Stress Disorder Short Scale (NSESSS) scale description of the individual items.

	Never/rarely	Sometimes/most of the time
Having “flashbacks,” that is, you suddenly acted or felt as if a stressful experience from the past was happening all over again (for example, you reexperienced parts of a stressful experience by seeing, hearing, smelling, or physically feeling parts of the experience)?	16.944 (81.81%)	3.768 (18.19%)
Feeling very emotionally upset when something reminded you of a stressful experience?	11.931 (57.60%)	8.781 (42.40%)
Feeling detached or distant from yourself, your body, your physical surroundings, or your memories?	16.719 (80.72%)	3.993 (19.28%)
Trying to avoid thoughts, feelings, or physical sensations that reminded you of a stressful experience?	13.440 (64.89%)	7.272 (35.11%)
Being "super alert,” on guard, or constantly on the lookout for danger?	16.083 (77.65%)	4.629 (22.35%)
Feeling jumpy or easily startled when you hear an unexpected noise?	14.515 (70.08%)	6.197 (29.92%)
Being extremely irritable or angry to the point where you yelled at other people. Got into fights or destroyed things?	16.271 (78.56%)	4.441 (21.44%)

Patients with pre‐existing mental disorders reported higher levels of traumatic symptoms in terms of global score at NSESSS (9.47 ± 5.97) compared to health care professionals (5.44 ± 4.65). These univariate differences were therefore tested in a regression model, weighted for the propensity score. According to the multivariate regression models, younger people (aged 18–25‐year old) tended to report higher levels of traumatic symptoms, compared with other age groups of participants (*B* = 2.89, 95% confidence interval, CI = 2.28–3.5, *p* < .0001), even after controlling for the severity of depressive, anxiety, and stress symptoms as well as the general health status (Table 3).

**TABLE 3:** Predictors of severity of traumatic score at National Stressful Events Survey Acute Stress Disorder Short Scale (NSESSS) in the global sample (*N* = 20,720).

	B	CI 95%	*p*‐Value
Mental disorder. Yes	2.57	2.04–3.09	.000
Health care professional	.37	.02–.72	.036
COVID+	−.75	−1.30 to −.20	.007
Physical comorbidities. Yes	.14	.0190704 .7175616	.424
Geographical region. Ref. Southern			
Northern	.5670742	.2858589 .8482896	.000
Central	.12442518	−1.204786 1.116282	.829
Islands	1.193218	.8539669 1.452469	.000
Week. Ref. first week of May 2020			
Week 23 April–29 April	−.222263	−.8307521 .3862261	.270
Week 16 April–22 April	−.2615754	−.8931254 .3699747	.116
Week 9 April–15 April	−.4939765	−1.058711 .0707584	.020
Week 30 March–8 April	−.4003884	−.9098876 .1091107	.075
Avoidant_coping	.0817328	.0161748 .1472909	.014
Approach_coping	.0113131	−.0370639 .0596902	.702
GHQ_method bin_tot	.1240407	.0520041 .1960772	.001
Connor_tot	.0044595	−.0080187 .0169376	.284
ISI_bin	3.332755	3.086142 3.579369	.000
UCLA_tot_mean	.3053827	.0636222 .5471432	.015
SIDAS_TOT	.0036599	−.0141069 .0214267	.550
Cases COVID	.0001382	−.0001221 .0003986	.298
Death COVID	.0003703	−.0014601 .0022007	.692
Pharmacological treatment	.185215	−.1772426 .5476727	.317
Constant	2.669351	1.206173 4.1325	.000

*Note*: Regression model has been controlled for individual factors, such as age and gender.

Abbreviations: CI, confidence interval; GHQ, General Health Questionnaire; ISI, Insomnia Severity Index; SIDAS, Suicidal Ideation Attributes Scale.

Furthermore, those affected by pre‐existing mental disorders had a higher risk of presenting acute traumatic symptoms with beta coefficient (B) of 2.57 (95% CI = 2.04–3.10; *p* < .0001), whereas those with pre‐existing physical health conditions did not present a higher risk of developing acute traumatic symptoms.

Other significant predictors included the presence of insomnia/sleep disorder (*B* = 3.32, 95% CI = 3.08–3.57) and being female (*B* = 1.49, 95% CI = 1.22–1.75) (Table 3).

## 5 DISCUSSION

Stressful life events, such as natural disasters (Carmassi et al., 2020, 2021; Dell'Osso et al., 2011), wars (Kang et al., 2019; Patton et al., 2021; Taylor et al., 2020), pandemics (Alberque et al., 2022; Chau et al., 2021; Delanerolle et al., 2022: Hassan et al., 2022; Volpe et al., 2022), represent indisputable triggers for the onset of acute stress or post‐traumatic stress symptoms in exposed people, including hyperarousal, reexperiencing of the stressful event or intruding memories, and flashbacks which could lead to a global functional impairment and complications as maladaptive behaviors or increased risk of suicidal attempts (American Psychiatric Association, 2013). The increased alarm due to the worldwide pandemic and the direct effects of lockdown measures led to a significant increase in the perception of the risk of contagion and, consequently, a constant feeling of threat. Peritraumatic distress following exposure to a traumatic event has been recognized as a significant predictor for the development of posttraumatic stress symptomatology (Candel & Merckelbach, 2004; Megalakaki et al., 2021; Pollice et al., 2012; Solomonov et al.,^2022^).

In general, individuals who had experienced acute stress symptomatology have an increased risk of developing PTSD within few months after the event. The severity of ASD can also significantly predict the severity of PTSD symptoms (Bryant, 2022; Carr et al., 1997; Li et al., 2021). This has also been confirmed for acute stress symptomatology due to the COVID‐19 pandemic (Shahrour & Dardas, 2020; Ye et al., 2020) and could also be indicative for the sample included in the present study as a predictive risk factor for such individuals.

Based on such premises, the COMET study was the first trial evaluating the global impact of the COVID‐19 pandemic and its related containment measures on mental health and well‐being in a wide sample of the Italian population (Giallonardo et al., 2020). Specifically, the purpose of the present study was to investigate how the lockdown and containment measures issued in Italy during the first wave of the Sars‐Cov‐2 virus impacted on the occurrence of acute stress symptoms in the general population and to evaluate the different levels of acute symptoms in those with preexisting mental health problems compared with health care professionals and those directly infected by the COVID‐19.

Some positive predictors of the severity of acute stress symptomatology were found. A preexisting mental disorder condition was found to be a condition that was associated with higher severity of symptomatology, as expected. A preexisting mental disorder condition might represent a potential condition associated with reduced ability to cope appropriately with stressful situations. In fact, according to the stress–vulnerability theory by Zubin and Spring, people suffering from mental disorders can experience a worsening of clinical symptoms or a new acute episode due to the exposure to traumatic/stress factors. This is as true for patients as it is for health care workers; in fact, this category of workers showed significantly higher scores than the rest of our sample. As largely known from the literature, health care workers present a higher risk of developing acute and/or post‐traumatic stress symptoms because their close‐contact with COVID‐19 patients and the daily and repeated closer experience of the effects of the virus during their work activities in hospitals and care settings (Andhavarapu et al., 2022; Bayazit et al., 2022; Schou‐Bredal et al., 2022; Baker et al., 2022).

We found that the geographical areas showing the worst trend about acute stress symptomatology during the lockdown period were the Northern regions and the Islands. In particular, Northern Italy was affected by a higher severity of the spread and worst viral symptomatology which resulted in an earlier intensification of containment measures and an earlier and greater alarm, compared to the rest of Italy (Di Girolamo et al., 2022). It is also interesting to note that our data confirm the hypothesis of an increase of acute stress symptoms with respect to the containment measures. In fact, our results suggest a negative effect on mental health which should be due to the lockdown measures especially in the middle phase of restrictive measures (in the week from April 9th to April 15th) over the 4 weeks of examination. This trend was followed by a progressive improvement of symptoms just before the easing of containment measures, confirming that the high levels of acute stress symptoms trended directly with the social isolation measures due to the pandemic.

Finally, another interesting finding is that avoidant coping strategies were correlated with the onset and severity of acute stress symptomatology. This represents a relevant finding; in fact, the presence of avoidant symptoms seems to be a nuclear component of maintaining a state of increased alertness: the avoidance and lack of exposure of the individual fuels a higher risk perception and thus a higher presence of stress related symptoms. Lastly, we showed that sleep disorders and social isolation also represent positive predictors for acute stress; this would thus corroborate the hypothesis that subjective feelings of loneliness and social isolation as well as insomnia are associated with a worsening of acute stress symptomatology (Cleper et al., 2022; Dell'Osso et al., 2022; Li et al., 2020; Morin et al., 2021; Pappa et al., 2020).

When discussing our results, some limitations should be considered. Due to lockdown measures, it was possible to carry out the survey exclusively through an anonymous online platform. This format could have biased the sample limiting elderly people or those living in socially disadvantaged contexts. Moreover, we asked participants whether they had any previous mental and/or physical disorder, but we did not confirm the diagnosis by a physical or a mental reexamination, nor we used a structured assessment tool such as the SCID‐5. Furthermore, the survey was carried out on Internet, and it was not possible to double check participants’ geographical location.

Differences in the levels of acute traumatic symptoms among people with pre‐existing mental health problems, health care professionals and those infected by COVID were evaluated in order to describe the possible influence of those conditions on the experience of traumatic symptoms. However, such differences should be further confirmed in long‐term studies, using several follow‐up assessment points and structured assessment tools administered by expert clinicians. In fact, it should be considered that preexisting mental health conditions could be present also in health care workers. Therefore, further studies are needed for clarifying the complex interaction among different moderator variables.

Another limitation is the use of a proxy measure for evaluating the exposure to the traumatic event, that is, the lockdown measures. It should be considered that beyond the lockdown measures, several other potential traumatic stressors could have impacted psychological distress symptoms. The cross‐sectional design of the survey allowed only a single time assessment and does not provide any causal relationship between the exposure to the lockdown and the presence of traumatic symptoms.

Indeed, our study had some strengths to consider. First, the large sample: this is one of the first studies carried out in different geographic Italian regions with a large sample of the general population during the lockdown period. Second, validated and reliable assessment tools were used to characterize acute stress symptoms and the impact of the event on the sample.

## 6 CONCLUSIONS

In conclusion, the COVID‐19 pandemic had a huge impact on the lifestyle and mental health of the general population worldwide (Geddes, 2021; Gorwood & Fiorillo, 2021; Sampogna et al., 2022). The growing concern about the risk of contagion as well as the measures of social isolation generated by the lockdown had important implications, such as the appearance of hyperarousal and other acute stress symptoms in the general population, especially in those which have characteristics of vulnerability such as a preexisting mental disorder or kinds of occupation as for the health care workers (Berry, 2021; Geddes, 2021; Schomerus et al., 2021). Symptoms of acute distress could positively predict for the occurrence of PTSD; therefore, future studies are needed to evaluate the long‐term impact of containment measures on that kind of individuals (Reed et al., 2022).

## AUTHOR CONTRIBUTIONS

All authors have actively contributed to the manuscript and approved the final version. C. Carmassi and G. Sampogna share first authorship.

## CONFLICT OF INTEREST STATEMENT

The authors declare no conflicts of interest.

## FUNDING INFORMATION

This research received no external funding.

### PEER REVIEW

The peer review history for this paper is available at https://publons.com/publon/10.1002/brb3.3314

## INFORMED CONSENT STATEMENT

Informed consent was obtained from all subjects involved in the study.

## Data Availability

Data are available upon request to corresponding author.
